# Decreased nonvertebral fracture incidence with zoledronate use for >3 years: post hoc analysis of a Randomized Controlled Trial

**DOI:** 10.1093/jbmr/zjag012

**Published:** 2026-01-22

**Authors:** Ian R Reid, Anne M Horne, Borislav Mihov, Sonja Bastin, Mark J Bolland, Gregory D Gamble

**Affiliations:** Department of Medicine, Faculty of Medical and Health Sciences, University of Auckland, Auckland, 1142, New Zealand; Departments of Endocrinology and Radiology, Auckland District Health Board, Auckland, 1023, New Zealand; Department of Medicine, Faculty of Medical and Health Sciences, University of Auckland, Auckland, 1142, New Zealand; Department of Medicine, Faculty of Medical and Health Sciences, University of Auckland, Auckland, 1142, New Zealand; Departments of Endocrinology and Radiology, Auckland District Health Board, Auckland, 1023, New Zealand; Department of Medicine, Faculty of Medical and Health Sciences, University of Auckland, Auckland, 1142, New Zealand; Departments of Endocrinology and Radiology, Auckland District Health Board, Auckland, 1023, New Zealand; Department of Medicine, Faculty of Medical and Health Sciences, University of Auckland, Auckland, 1142, New Zealand

**Keywords:** osteoporosis, anti-resorptives, bisphosphonates, zoledronate, zoledronic acid

## Abstract

The anti-fracture efficacy of most osteoporosis drugs is assessed in trials of up to 3 yr duration. However, treatment of osteoporosis is required long-term, so it is important to know whether effectiveness of treatment changes over time. To date, this information has only been available from open extensions of the treatment groups from clinical trials. Such data are subject to selective loss of frailer patients from the cohort, and no placebo comparator group is available to permit reliable measurement of anti-fracture efficacy. The 6-yr Auckland zoledronate RCT administered zoledronate or placebo every 18 mo to 2000 osteopenic women aged >65 yr. It is longer than most osteoporosis trials, and only 3.6% of participants withdrew or were lost to follow-up. Therefore, anti-fracture efficacy can be validly compared between the first and second halves of this study. There were 178 non-vertebral fractures in the placebo group and 108 in the zoledronate group (excluding fractures of the hands, feet, and face). Fracture rates per 1000 women-years in the placebo group were 28 (95% CI 23, 35) and 32 (26, 40) in years 1-3 and years 4-6, respectively. In the zoledronate group, fracture rates were 23 (18, 30) and 13 (9, 18) per 1000 women-years in the first and second halves of the study. The rate ratios for non-vertebral fractures were 0.83 (0.60, 1.14) in years 1-3, and 0.40 (0.27, 0.58) in years 4-6 (between-period comparison, *p* < .05). Rate ratios for total fragility fractures, which include vertebral fractures also, showed a similar pattern: years 1-3, 0.76 (0.56, 1.02) and years 4-6, 0.39 (0.29, 0.53). These findings suggest that the efficacy for non-vertebral fracture prevention of anti-resorptive drugs has been underestimated because of the short-term evaluation of their effects. Further, it indicates that longer term treatment with these drugs may yield greater benefit to patients.

## Introduction

The anti-fracture efficacy of most osteoporosis drugs is assessed in trials of up to 3 yr. This trial duration has been chosen on pragmatic grounds, since it is the minimum time necessary to establish anti-fracture efficacy, a prerequisite for drug registration. Current drugs have substantial efficacy against vertebral fractures but prevention of non-vertebral fractures has been disappointing, often only about 20%.^1^ Many fracture studies have had subsequent trial extensions, but in these the placebo group is usually discontinued or crossed over to active treatment, for ethical or pragmatic reasons. Accordingly, there are very few data from randomized controlled trials documenting the longer term anti-fracture efficacy of the treatments we currently use. It is quite possible that efficacy could vary with longer term use, either positively or negatively, or that it could follow different time-courses at different skeletal sites, since bone turnover is substantially different between cortical and trabecular bone. If this were the case, then it would be highly relevant to the determination of the optimal duration of treatment. A change in anti-fracture efficacy over time would also impact on pharmaco-economic analyses and, thus, on the appropriate use of the drug at various baseline fracture risk levels.

Our study of zoledronate in osteopenic women aged over 65 yr,^2^ in which >96% of participants provided outcome data to 6 yr, provides a unique opportunity to determine whether anti-fracture efficacy compared with placebo varies over a much longer period than has been examined previously.

## Materials and Methods

The details of the original study protocol have already been published,^2^ but are summarized here. The study was registered at the Australian New Zealand Clinical Trials Registry, number ACTRN12609000593235.

### Study design

This was a prospective, randomized, placebo-controlled, double-blind trial to determine the anti-fracture efficacy of zoledronate in osteopenic postmenopausal women. A total of 2000 women were recruited using electoral rolls and randomized (1:1) to receive 4 infusions of either zoledronate 5 mg or normal saline at 18-mo intervals. Each participant was followed for 6 yr. Calcium supplements were not supplied. Trial procedures took place at the Clinical Research Centre, University of Auckland, with participant visits every 18 mo. Calculations of risk of hip or osteoporotic fracture have been made using FRAX (https://www.sheffield.ac.uk/FRAX/index.aspx). The trial was approved by the regional Health and Disability Ethics Committee, and all participants provided written informed consent. In the zoledronate group, there was an open extension of the study to 10 yr without further administration of study medication.^3^

Participants recorded fractures in a quarterly questionnaire and were also asked to report fractures immediately by telephone. Symptomatic fractures were confirmed from radiology reports, or by review of radiographs (by S.B. or I.R.R.) if reports were equivocal. Lateral spine radiographs were obtained at baseline, 3 and 6 yr for vertebral morphometry. Digital images were assessed semi-quantitatively by a radiologist (S.B.), as described by Genant.^4^ Where this assessment suggested an incident fracture, vertebral body heights were measured. A decrease of ≥20% and ≥4 mm defined an incident fracture. Symptomatic vertebral fractures were confirmed on vertebral morphometry assessment. Pathological fractures were excluded from all trial endpoints.

The primary endpoint of the original study was fragility fracture, comprised of non-vertebral fractures (excluding toes, metatarsals, fingers, metacarpals, skull, facial bones, and mandible) and radiographic vertebral fractures. Trauma severity was not part of the definition of fragility fracture.

### Participants

Participants were ambulant postmenopausal women aged >65 yr, with T-score at either the right or left total hip or femoral neck in the range −1.0 to −2.5, who were able to give informed consent. Exclusion criteria were: LS T-score <−3.0, eGFR <30 mL/min, major systemic disease, malignant disease in the last 2 yr, metabolic bone disease, or regular use of bone-active drugs in the previous year (including estrogen, anti-estrogens, and systemic glucocorticoids in doses equivalent to prednisone ≥2.5 mg/day).

### Statistical analysis

Fractures per 1000 women years were estimated using www.openepi.com with CIs calculated by the Mid-P exact method. The analysis included all fractures occurring during each triennium, not just first fractures. Generalized estimating equations analysis was used to model the interaction between fracture rates in each time epoch and treatment allocation (assuming a Poisson distribution, log link function and log duration of follow-up as offset) using Proc Genmod (SAS v 9.4, SAS Institute Inc.).

Neither the Health Research Council of New Zealand nor Novartis had any role in study design, data collection, analysis, interpretation of data, report writing, or in the decision to submit the paper for publication.

## Results

**Figure 1:**
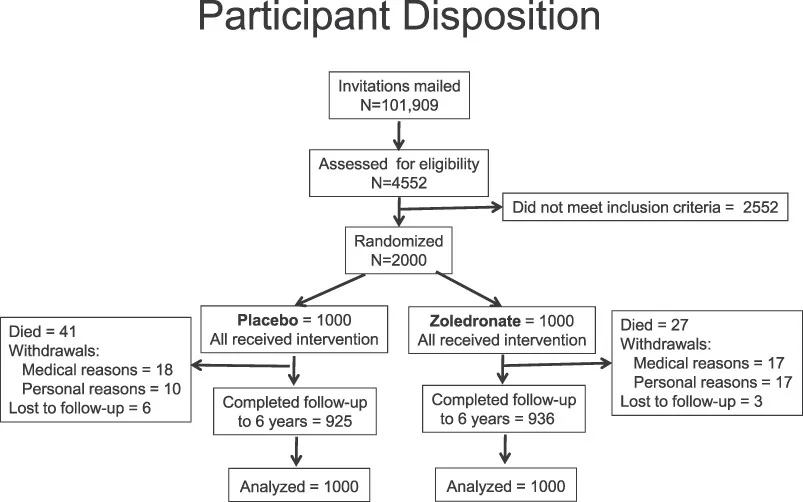
Enrolment, randomization, and follow-up of trial participants. Participants described as completing follow-up to 6 yr, either attended the 6-yr visit, or had a telephone consultation that permitted ascertainment of fractures. In the placebo group, 825 women received 4 doses of the trial regimen, 80 received 3 doses, 53 received 2 doses, and 42 received 1 dose. In the zoledronate group, 806 women received 4 doses of the trial regimen, 58 received 3 doses, 50 received 2 doses, and 86 received 1 dose.

Clinical characteristics of the study subjects are set out in Table 1 and are consistent with an older postmenopausal cohort of women at only moderate risk of fracture. The flow of participants through the study in shown in Figure 1. Particularly relevant to the present analysis is that only 3.6% of participants either withdrew or were lost to follow-up, and these figures were comparable across the 2 study groups. Study follow-up was continued in those who stopped taking study medications or who were started on bone-active drugs by their own doctors.

**Table 1:** Baseline characteristics.

	**Placebo**	**Zoledronate**
***n***	1000	1000
**Age (yr)**	71 (5.1)	71 (5.0)
**European ethnicity**	940 (94%)	954 (95%)
**Height (cm)**	160.4 (5.8)	160.7 (5.8)
**Weight (kg)**	69.2 (12.2)	69.1 (12.5)
**BMI (kg/m** ^**2**^**)**	26.9 (4.7)	26.8 (4.6)
**Dietary calcium intake (mg/day)**	882 (388)	871 (360)
**History of non-vertebral fracture after age 45 yr**	238 (24%)	237 (24%)
**Prevalent vertebral fracture**	126 (13%)	137 (14%)
**FRAX 10-yr osteoporotic fracture risk (%)**	12.0 (9,15)	12.0 (9,16)
**FRAX 10-yr hip fracture risk (%)**	2.3 (1.5, 3.8)	2.4 (1.5, 3.9)
**Bone density T-scores**		
**Lumbar spine**	−0.87 (1.16)	−0.91 (1.12)
**Total hip**	−1.24 (0.60)	−1.27 (0.59)
**Current smokers (%)**	33 (3.3%)	23 (2.3%)

Data are mean (SD) or number (%), except for FRAX risks which are median (interquartile range).

**Figure 2:**
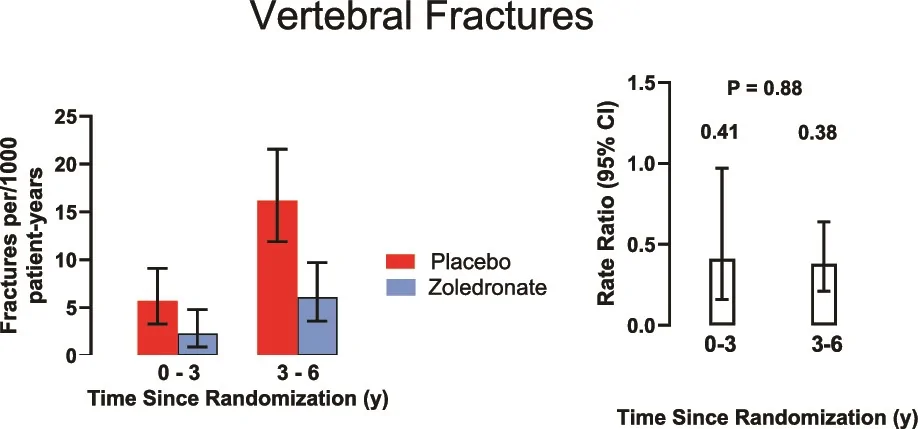
Rates of vertebral fractures and the resulting rate ratios comparing placebo and zoledronate groups, in the first and second triennia of a 6-yr trial in osteopenic women aged >65 yr. The right-hand panel compares the rate ratios between the treatment groups in the first and second halves of the trial.

Fracture data from the first and second halves of the study are set out in Table 2. In the placebo group, vertebral fracture rates increased almost 3-fold between the first and second triennia. In the zoledronate group, vertebral fracture rates were much lower, but also increased between 0-3 yr and 3-6 yr, so the rate ratios between the treatment groups were similar across the 2 periods (Figure 2). As shown in our original publication from this study,^2^ most vertebral fractures were detected at years 3 and 6 when the scheduled vertebral radiographs were carried out.

**Table 2:** Fracture data in first and second triennia of trial.

**Fracture category**	**Time**	**Placebo**			**Zoledronate**			**Rate ratio (95% CI)**
		**Women with fracture (*n*)**	**Fractures (*n*)**	**Fractures per 1000 woman-yr (95% CI)**	**Women with fracture (*n*)**	**Fractures (*n*)**	**Fractures per 1000 woman-yr (95% CI)**	
**Vertebral**								
	0-3 yr	15	17	5.7 (3.3, 9.1)	6	7	2.3 (0.9, 4.8)	0.41 (0.16, 0.97)
	3-6 yr	36	47	16.2 (11.9, 21.57)	17	18	6.1 (3.6, 9.7)	0.38 (0.21, 0.64)
**Nonvertebral**								
	0-3 yr	75	84	28.2 (22.5, 34.9)	65	70	23.4 (18.2, 29.6)	0.83 (0.60, 1.14)
	3-6 yr	81	94	32.3 (26.1, 39.5)	38	38	12.9 (9.1, 17.7)	0.40 (0.27, 0.58)
**Fragility**	0-3 yr	87	101	33.9 (27.6, 41.2)	71	77	25.7 (20.3, 32.2)	0.76 (0.56, 1.02)
	3-6 yr	114	141	48.5 (40.8, 57.2)	54	56	19.0 (14.4, 24.7)	0.39 (0.29, 0.53)

**Figure 3:**
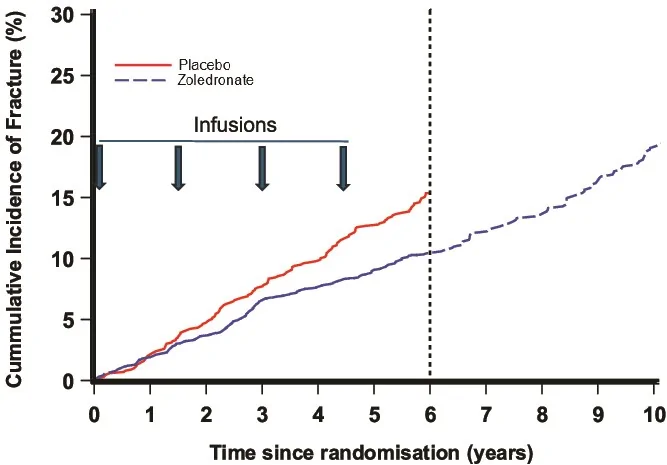
Cumulative incidence of non-vertebral fracture during the 6-yr, randomized, placebo-controlled trial was calculated with the use of the Cox proportional-hazards model for the risk of first non-vertebral fragility fracture (excluding fractures of the toes, metatarsal bones, fingers, metacarpal bones, skull, facial bones, and mandible). Non-vertebral fracture data are also shown in the zoledronate group during the observational extension to 10 yr, during which no study medications were administered. The vertical arrows indicate the timing of the zoledronate infusions. Adapted from Reid et al.,^3^ used with permission from Elsevier.

**Figure 4:**
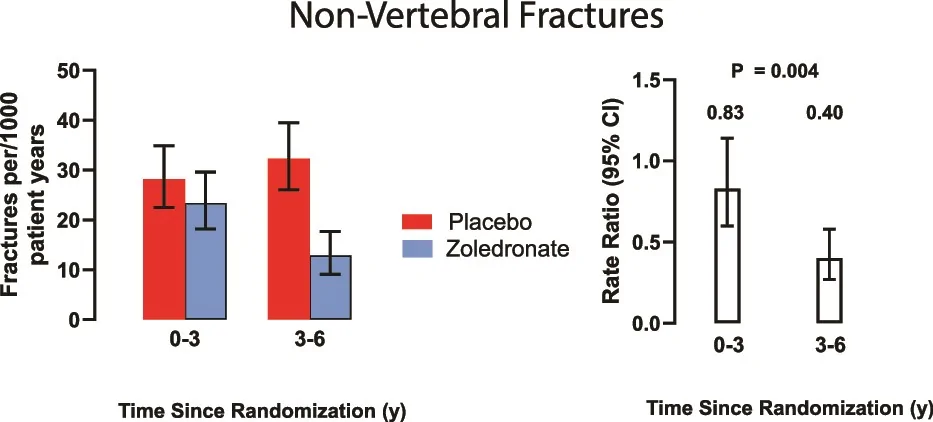
Rates of non-vertebral fractures and the resulting rate ratios comparing placebo and zoledronate groups, in the first and second triennia of a 6-yr trial in osteopenic women aged >65 yr. The right-hand panel compares the rate ratios between the treatment groups in the first and second halves of the trial.

The cumulative incidence of non-vertebral fractures throughout the study (and in the open extension to 10 yr) is shown in Figure 3. In the placebo group, the fracture rate was stable across the study period, as also indicated by the fracture rates shown in Table 2. In contrast, the plot of the zoledronate group suggests an inflection at about 3 yr, consistent with the reduction in non-vertebral fracture rate by almost one half between the first and second triennia shown in Table 2. As a result, the rate ratio for non-vertebral fractures fell from 0.83 (0.60, 1.14) in 0-3 yr to 0.40 (0.27, 0.58) in 3-6 yr (*p* = .004 for difference between rate ratios) (Figure 4). Rate ratios for total fragility fractures, which include vertebral fractures also, showed a similar pattern: years 1-3, 0.76 (0.56, 1.02); years 4-6, 0.39 (0.29, 0.53).

## Discussion

This post hoc analysis of a 6-yr randomized trial of zoledronate in older osteopenic women, clearly shows that the efficacy of zoledronate in preventing non-vertebral fracture rates increased substantially between the first and second halves of this study, whereas efficacy of vertebral fracture prevention was stable over this period. The hypothesis addressed in these analyses was formed following the conclusion of the trial, so it may be a chance finding. However, it is robustly statistically significant, and the lower non-vertebral fracture rate is very consistent from the end of year 3 to the end of year 6, and indeed for several more years after that in the study extension even though there was no zoledronate administration past four and a half years.^3^ This is a potentially important finding, since the 60% reduction in non-vertebral fracture rate seen in years 3-6 would place zoledronate in the first rank of anti-osteoporosis medications. This suggests that the longer-term efficacy of this medication has been substantially underestimated and that its cost-effectiveness might be even greater than has been estimated previously.^5^

The difference in time-courses of vertebral and non-vertebral anti-fracture efficacy of zoledronate revealed in the present analysis is not unexpected, because vertebral bone is predominantly trabecular, and therefore has higher bone turnover. As a result, its uptake of bisphosphonates is greater and its bone density response to anti-resorptive drugs is more rapid. Accordingly, it is not surprising that >3 yr might be needed to achieve optimal anti-fracture efficacy at predominantly cortical bone sites. This could impact on determining the optimal regimen for the use of this drug. Outside the present study, there are few controlled data documenting the effects of zoledronate on non-vertebral fractures beyond 3 yr, since in the phase 3 study program only 921 completed follow-up to 6 yr, and the original placebo group was discontinued at 3 yr. Many experts recommend zoledronate annually for 3 doses, based on the phase 3 program,^6^ resulting in the last dose being given 2 yr after the first. That regimen achieved a lesser prevention of non-vertebral fracture (hazard ratio 0.75 [0.64, 0.87])^7^ than the present regimen did between 3 and 6 yr (see Table 2), suggesting that longer term treatment is more effective.

Time trends in anti-fracture efficacy in the two phase three trials of zoledronate can be assessed from the published Kaplan–Meier plots.^7^^,^^8^ The slopes of those plots suggest that greater reductions in fracture rates were achieved in the second half of those 3-yr studies. The study of Black et al. discontinued the placebo group at the end of year 3 but continued some patients from the zoledronate group, half randomized to continue zoledronate and half to cross-over to placebo.^9^ Vertebral fracture rates increased in those crossed over to placebo, but both groups in the extension maintained the lower non-vertebral fracture rate achieved during the core study. Since fracture rates would be expected to increase with advancing age, the absence of such a trend suggests that, were placebo data available from years 4-6, increasing anti-fracture efficacy would have been evident. Further analysis of our recent 10-yr study of very infrequent zoledronate in early postmenopausal women^10^ also demonstrates increasing anti-fracture efficacy in the second half of the study compared with the first.^11^ The apparent consistency of this finding across the present study and those of Black, Lyles, and Bolland^7^^,^^8^^,^^10^ suggests that this is generalizable across a wide range of ages and fracture risks.

There are few data describing the long-term trends in anti-fracture efficacy of other bisphosphonates. The alendronate phase 3 trial was placebo-controlled to 3 yr but fracture data from some of the alendronate group were published up to 10 yr.^12^^,^^13^ These suggested that non-vertebral fracture risk was reduced by one-third in those on long-term alendronate compared with a placebo group adjusted for age, whereas fracture risk reduction at 3 yr was 20%. However, the small number of patients at 10 yr makes this estimate unreliable. The Fracture Intervention Trial of alendronate showed no change in risk of clinical fractures in the first 15 mo, but over the subsequent 3 yr the cumulative proportions with fracture did diverge.^14^ The Fracture Intervention Trial was extended into a further 5-yr study, FLEX. Detailed data permitting comparison of fracture rates between the two studies are not published, but the FLEX paper states that non-vertebral fracture incidence was similar in alendronate groups of the 2 studies.^15^ As discussed for the phase 3 alendronate study, a persisting reduced fracture rate in alendronate users combined with the expectation that rates would have increased over time had the placebo group been continued, could be consistent with increasing efficacy compared with placebo over time.

Superficially, similar data to the present analysis have been presented from the extension to 10 yr of the FREEDOM study of denosumab.^16^ The core FREEDOM study reported a hazard ratio for non-vertebral fracture of 0.80 (0.67, 0.95) but the reported rates of non-vertebral fractures fell by a further 40% in the first 2 yr of the extension. However, the study extension admitted only compliant patients and there was a loss of 40% of the cohort going into the extension. Only one-third of the denosumab cohort completed follow-up to 10 yr, and there is likely to have been selective loss of frailer patients over this period. These problems and the absence of the placebo group past year 3 mean that denosumab’s long-term anti-fracture efficacy cannot be determined, though the similarity of those findings to the present results suggests that increasing efficacy over time might be a general property of anti-resorptive drugs.

The analysis presented here strongly suggests that the efficacy of zoledronate for the prevention of non-vertebral fractures increased between the first and second triennia of our recent placebo-controlled trial of this medicine. The other published trials of zoledronate appear to be consistent with this finding, and a similar claim has been made for denosumab, though those data are uncontrolled and have substantial loss of study participants over the follow-up period. It is highly desirable that the question of the evolution of efficacy over-time should be addressed in future studies of anti-osteoporosis medications, though the logistical and financial obstacles along this path are substantial. If anti-fracture efficacy does change over time, then this could be dose-dependent and could differ between agents. It has major implications for the estimation of cost-effectiveness of therapies, for ascertaining their comparative efficacies, and for determining the optimal duration of their use. The present analysis has immediate implications for the optimal use of zoledronate, since it indicates that 18-monthly administration of this drug over >3 yr achieves an efficacy for non-vertebral fractures that is substantially greater than the annual administration of 3 doses of this drug, a regimen which continues to be widely used.

## Data Availability

The data that support the findings of this study are available from the corresponding author upon reasonable request.
